# SARS-CoV-2 Subgenomic N (*sgN*) Transcripts in Oro-Nasopharyngeal Swabs Correlate with the Highest Viral Load, as Evaluated by Five Different Molecular Methods

**DOI:** 10.3390/diagnostics11020288

**Published:** 2021-02-12

**Authors:** Massimo Zollo, Veronica Ferrucci, Barbara Izzo, Fabrizio Quarantelli, Carmela Di Domenico, Marika Comegna, Carmela Paolillo, Felice Amato, Roberto Siciliano, Giuseppe Castaldo, Ettore Capoluongo

**Affiliations:** 1 CEINGE, Biotecnologie Avanzate, 80131 Naples, Italy; massimo.zollo@unina.it (M.Z.); veronica.ferrucci@libero.it (V.F.); barbara.izzo@unina.it (B.I.); quarantelli@ceinge.unina.it (F.Q.); didomenico@ceinge.unina.it (C.D.D.); marika.comegna@unina.it (M.C.); felice.amato@unina.it (F.A.); sicilianor@ceinge.unina.it (R.S.); giuseppe.castaldo@unina.it (G.C.); 2 Dipartimento di Medicina Molecolare e Biotecnologie Mediche, Università di Napoli Federico II, 80138 Naples, Italy; 3 Department of Medicina di Laboratorio e Trasfusionale, AOU Federico II, 80138 Naples, Italy; 4 Dipartimento di Clinica e Medicina Sperimentale, Università degli Studi di Foggia “Emanuele Altomare” Via Napoli, 121, 71122 Foggia FG, Italy; carmela.paolillo@gmail.com

**Keywords:** SARS-CoV-2 subgenomic regions, viral load, CE-IVD

## Abstract

The COVID-19 pandemic has forced diagnostic laboratories to focus on the early diagnostics of SARS-CoV-2. The positivity of a molecular test cannot respond to the question regarding the viral capability to replicate, spread, and give different clinical effects. Despite the fact that some targets are covered by commercially-available assays, the identification of new biomarkers is desired in order to improve the quality of the information given by these assays. Therefore, since the subgenomic transcripts (*sgN* and *sgE*) are considered markers of viral activity, we evaluated these subgenomic transcripts in relation to the genomic amplification obtained using five different commercial CE-IVD tools. **Methods**: Five CE-IVD kits were compared in terms of their capability to detect both synthetic SARS-CoV-2 viral constructs (spiked in TMB or PBS medium) and targets (*N*, *E*, *RdRp* and *Orf1ab genes*) in twenty COVID-19–positive patients’ swabs. The *sgN* and *sgE* were assayed by real-time RT-qPCR and digital PCR. **Results**: None of the diagnostic kits missed the viral target genes when they were applied to targets spiked in TMB or PBS (at dilutions ranging from 100 pg to 0.1 pg). Nevertheless, once they were applied to RNA extracted from the patients’ swabs, the superimposability ranged from 50% to 100%, regardless of the extraction procedure. The *sgN* RNA transcript was detected only in samples with a higher viral load (Ct ≤ 22.5), while *sgE* was within all of the Ct ranges. **Conclusions**: The five kits show variable performances depending on the assay layout. It is worthy of note that the detection of the *sgN* transcript is associated with a higher viral load, thus representing a new marker of early and more severe infection.

## 1. Introduction

The coronavirus disease 2019 (COVID-19) outbreak has rapidly involved all countries, resulting in the ongoing pandemic [1,2]. The global pandemic still occurs, with continuous issues related to the absence of high-throughput and rapid technologies that are able to deliver rapid diagnostic tests on large-scale populations. Therefore, the reagents and chemicals that are now working on the main hospital routine platforms are generally based on low–medium throughput technologies, with there sometimes being a restriction in the reagent recovery, and market saturation for those working on automated pipelines and higher throughput platforms [2]. Our lab has been recognized as one of the sixteen accredited centers for molecular diagnostics of COVID-19 infection, and we participated in the Campania region screening as a part of the regional laboratory network, namely ‘Campania Coronet’. 

However, since the market saturation did not make available large numbers of kits, reagents, chemicals and disposables [2,3,4], we were forced to use different tools in order not to stop our diagnostic activity, in compliance with the agreement with the regional task force for SARS-CoV-2 screening. However, many practical and technical issues frequently arise in laboratories performing COVID-19 molecular assays, particularly regarding the nucleic acid extraction, nucleic acid amplification reagents, and interpretation of test results [5,6].

Therefore, from among the most reliable and effective CE-IVD kits recommended by the Italian health authority (Istituto Superiore di Sanità; ISS) (https://www.trovanorme.salute.gov.it/norme/renderNormsanPdf?anno=2020&codLeg=73799&parte=1%20&serie=null (accessed on 4 February 2021)), we selected three out of twelve reagents: Allplex 2019-nCov Assay (SEEGENE: kit-A), BOSPHORE-V2 Novel Coronavirus 2019-Ncov (Anatolia: kit-B), and Realquality RQ-2019-NCOV (AB ANALITICA: kit-C), also following the literature reports [7,8,9]. Although the diagnostic market provides lots of solutions, Guglielmi [10] stated that the current PCR-based assays can test whether someone is infectious, but does not distinguish between individuals who carry the virus and those who are not likely to spread it. In fact, the viral load peaks early in SARS-CoV-2 infections and then gradually declines, with small amounts of virus RNA staying in the nasopharyngeal trait for weeks, or sometimes months [10]. Thus, it is very difficult to distinguish individuals with active and higher viral loads through the commonly-used routine tests. Nonetheless, in COVID-19 assay-positive individuals, who are screened for the first time and show these high Ct values for the detected targets, it is not possible to determine whether they are initial or previous infections [7,8]. In this context, we investigated whether other molecular transcripts of SARS-CoV-2 could provide useful information in terms of potential biomarkers of virus activity in this peculiar situation. Particularly, the transcriptome landscape of SARS-CoV-2 is also characterized by positive-sense sub genomic RNAs (sgRNAs) encoding for both structural and several accessory proteins [7,11]. SgRNAs are synthetized by nsp12 harbouring RNA-dependent RNA polymerase (RdRP) activity via a mechanism of discontinuous transcription according to the to the ‘leader–body fusion’ model [11]. Coronaviruses use these subgenomic RNA fragments, produced by discontinuous transcription, to translate the early proteins necessary for viral replication and encapsidation [12]: the ratios of the mean normalized sgRNAs per genome are estimated as being about 0.4% [12]. Kim et al. [11] reported that the *N* RNA is the most abundantly expressed transcript, followed by the *E*. Interestingly, as reported by Wölfel et al. [12], these viral sgRNAs are transcribed only in infected cells, and are not packaged into virions: therefore, they indicate the presence of actively-infected cells. Based on these findings, these authors [12] argued that, as high viral loads were always shown to be associated with SARS-CoV-2 isolation from early throat swabs, the sgRNAs could be considered as potential virus replication markers in the tissues of the upper respiratory tract [12]. In order to obtain proof of active virus replication in positive samples, Wölfel et al. [12] performed RT–qPCR tests to identify viral subgenomic mRNAs directly in clinical nasopharyngeal samples showing a decline of subgenomic RNAs from day ten. However, in throat swabs, all of the samples taken up to day five did not show any subgenomic mRNA detectability. Therefore, Wölfel et al. [12] stated that their findings indicate that the active replication of SARS-CoV-2 occurs in the throat during the first five days after the onset of symptoms. In support of these findings, we underline that Doddapaneni et al. [13] were able to obtain complete genomes (genomic and subgenomic regions) only on clinical samples with threshold cycle values <33. Consequently, the different performances of commercial kits could influence our capability both to detect and correlate the genomic viral load with the subgenomic expression. 

In this regard, we report herein the data gained on *sgN* and *sgE* transcripts assayed on oro-nasopharyngeal swab samples in relationship to genomic results obtained by using different kits for SARS-CoV-2 screening (namely, kit-A, kit-B and kit-C). We also evaluated, on the same samples, the performances of two newly-released kits provided by a commercial Korean company (Kit-D and Kit-E), which were not listed among those previously approved by the Italian Ministry. As already reported [1,6,7,8,9], most of diagnostic kits are based on the detection of SARS-CoV-2 genes encoding structural (e.g., spike protein [S], envelope protein [E], membrane protein [M], and nucleocapsid protein [N]) or non-structural (Orf1b/RdRp) proteins. We underline that, when the assay results are positive with a high threshold cycle (a Ct from 38 to 40, for example) for one single target (*N*, *E*, *RdRp*, *S*, *Orf1b*), an ambiguous interpretation is achieved. In this regard, particularly in the presence of lower viral loads [10], the kit cannot respond to all of the clinical diagnostic questions, such as: (a) does the positivity correspond to an active viral infection? (b) Does the subject have a potential infectious capacity? We aimed to verify the following issues: (a) are all of the kits superimposable in terms of the detection of the specific genomic targets detected? (b) Are the sgRNAs detected in the complete ranges of the threshold cycles? (c) Is there any potential to use subgenomic transcripts, in combination with viral genomic RNA, as ‘higher viral load surrogate markers’? (d) Can the transcription rate of subgenomic RNAs be related to the genomic target detected in the swab samples?

To this purpose, the sgRNAs encoding for Envelope protein (*sgE*) and nucleoprotein (*sgN*) were assayed through with different molecular approaches [11,12,13,14] on nasopharyngeal swabs collected from patients admitted to our hospital as being suspected for COVID-19 infection. The RT-qPCR has yet to be used as a method for the detection of SARS-CoV-2 subgenomic transcripts [12,15]. 

In summary, we show that sgRNAs (*sgN* and *sgE*) detection can provide useful information, to such an extent that we propose to use it in combination with routine assays to assess the early phase of infection in oro-nasopharyngeal swabs, it being the sgRNA *N* that is able to discriminate between higher and lower viral loads. 

## 2. Materials and Methods

### 2.1. Sample Collection

Oro-nasopharyngeal sampling was performed by means of commercial-flocked swabs collected in about 1 mL universal transport media (UTM (Copán, Brescia, Italy)) and sent to our Lab in boxes at a controlled temperature within four hours of the collection. The regional health department decided to centralize the swab drawing through a unique unit for samples collection, which consisting of skilled health professionals (coordinated by the ‘Istituto Zooprofilattico Sperimentale del Mezzogiorno’—IZSM) who were trained in performing the oro-nasopharyngeal swabs, which guaranteed homogeneity in the procedures surrounding the sample drawing. In this way, the standardization of the modality of the sample collection was obtained. Our study was approved by the Ethical Committee of Federico II University (n. of protocol 000576 of 10 April 2020), and was performed in compliance with the Declaration of Helsinki.

### 2.2. RNA Extraction

All of the samples compared in the present paper were extracted using an automated procedure on a MagPurix machine. In detail, a 200 µL volume was used to extract RNA by using a fully-automated system based on a MAGPURIX VIRAL/PATHOGEN NUCLEIC ACIDS kit (Zinexts, commercialized by Resnova, Genzano di Roma, Italy) running on a MAGPURIX 24 INSTRUMENT. MagPurix^®^ CE-IVD Reagent Kits are designed to provide the highest extraction quality through optimized protocols. All of the reagent kits are available in pre-packed environmentally-friendly boxes containing all of the components for 48 extractions: separable reagent cartridges, polygon reaction chambers, tip holders, single-sample tubes, elution tubes, filter tips and piercing pins, as well as additional specific buffer and an optional RNA carrier. No spiking-in with an internal control was performed on the starting swab, in order to obtain viral RNA that was free from RNAs that could potentially interfere with the different methods used. All of the RNA was eluted in 50 µL elution buffer provided by the manufacturers.

### 2.3. Amplification Kit Selection

Four CE-IVD reagent kits were used, following our Ministry of Health’s indications: the latter, in agreement with the recognized National Covid Reference Laboratories (Istituto Superiore di Sanità and Lazzaro Spallanzani Laboratory), provided a list of eleven kits recognized as being useful in SARS-CoV-2 routine diagnostics, see Table 1. 

The assays selected for the detection of genomic SARS-CoV-2 transcripts are reported in Table 2, in which all of the characteristics are listed in terms of the target gene, company producing or selling kit, instrumentations validated for the amplificon procedure, input of RNA, number of cycles, range of cycles indicative of positive results, limit of detection, and precision of the methods.

The performances of the three CE-IVD tools were also evaluated against the following new releases: the iONEBIO kit (Kit-D) and the GenePro COVID-19 ((Kit-E); Gencurix, SouthKorea), which has received CE-IVD and FDA clearance. The first was the only one working with a different chemistry, namely the loop-mediated isothermal amplification (LAMP) method, with positive results when the threshold cycle (Ct) values were below or equal to 25. 

In the first phase, we used artificial transcripts (see Section 2.4) to verify the performance of these kits. In a second phase, we switched the evaluation on twenty swabs previously analysed and confirmed as positive in two independent runs.

Following the manufacturers’ instructions, all of the samples were run in single. Nevertheless, in order to verify the performance of each kit, some samples were randomly run in duplicate, blind, pipetting the sample duplicate in different wells on each real-time plate. In this way, the instrument performance was also better evaluated in terms of the homogeneity of the running conditions of the thermal-block during the real-time PCR assay. The personnel should be maintained the templates and performed the run was chosen to be the same. This choice was taken in order to reduce biases related to the sample handling. 

### 2.4. Spike Oligonucleotides Synthesis

We reported, in Table 3, on RNA oligonucleotides (i.e., SARS-CoV-2_N_F1, 117mer; SARS-CoV-2_E, 113mer; Horizon; https://horizondiscovery.com (accessed on 4 February 2021)) which were synthetized by using the standard phosphoramidite solid-phase synthesis technology and 5’-hydroxyl (5’-SIL), in combination with an acid-labile orthoester on the 2’-hydroxyl (2’-ACE) as the protecting groups. In addition, the SARS-CoV-2_E oligonucleotide was O2’-methylated. Finally, the oligonucleotides were deprotected by removing the 2’-ACE protecting groups, desalted by ethanol precipitation, and purified with ion exchange high performance liquid chromatography (HPLC).

After this setting, we proceeded to spike-in the detection of both constructs by means of RNA serial dilutions, starting from 1 ng of the gene *N* and *E* constructs, obtaining the following dilutions: 1:10 (100 pg); 1:100 (10 pg); 1:1000 (1 pg); 1:10,000 (0.1 pg), as refereed to 1 ng (starting amount). The dilutions were performed by spiking in a TMB medium belonging to a negative sample that was previously tested in triplicate. The same setting was performed by using PBS as the dilution medium, in order to verify a possible matrix effect on the extraction procedure. All of the dilutions were run in triplicate.

### 2.5. Amplification of the Subgenomic Orf1 Region SgRNAs for N and E Regions Amplification

The oligonucleotide sequence of the primers is described in Table 4.

The total RNA was extracted from all of the positive samples. The sgRNA RT-PCR assay used the SuperScript IV VILO Master Mix (11756500, Invitrogen, Carlsbad, CA, USA), following the manufacturer’s instructions. The testing for sgRNAs used a leader-specific primer, as well as primers and probes targeting sequences downstream of the start codons of the *E* and *N* genes [11,13]. In addition, the SYBR-green technology was also used to detect the above subgenomic transcripits by a different couple of primers. In detail, the reverse transcription products (cDNA) were amplified by quantitative real-time PCR using a real-time PCR system (Quantstudio 5, Life Technologies, Monza MB, Italy). The target genes were detected using a Brightgreen 2× qPCR Mastermix low-rox (#Mastermix-lr; ABM.). These analyses were run using a PCR machine (Quantstudio 5) under the following conditions: hold stage, 50 °C for 2 min, 95 °C for 10 min; PCR stage, 95 °C for 15 s, 60 °C for 1 min (×60 cycles); melt curve stage, 95 °C for 15 s, 60 °C for 1 min; 95 °C for 15 s. The SYBR Green Primers are reported in Table 3.

The cDNA was obtained by random primer RT-PCR using the SensiFASTcDNA Synthesis Kit (Bioline, provided by Life Technologies Italia, Monza MB, Italy), using 5ul RNA extracted from nasopharyngeal swabs. We used the following primer setting (*sgN*-For AAACCAACCAACTTTCGATCTCTTGTA and *sgN*-Rev TCTGGTTACTGCCAGTTGAATC) to amplify the *sgN* region, and to perform the Sanger sequencing. All of the primer settings are reported in Figure S1 (supplementary files). The conditions for the target amplification were the following: cDNA was used as a template to amplify the *sgN* sequence by means of the Wonder Taq DNA Polymerase (EuroClone, Via Figino, 20/22 20016 Pero (MI)) according to the manufacturer’s instructions. The amplified products were extracted from agarose gel and purified with the Wizard SV Gel and PCR Clean-Up System kit (Promega). In brief, an equal volume of Membrane Binding Solution was added to the isolated fragment in order to allow the solubilization of the agarose; then, it was aliquoted in a silica column and centrifuged at 14,000 rpm for 1 min. The column was washed with 700 µL washing buffer and centrifuged again at 14,000 rpm for 1 min. In the final step, the DNA was eluted with Nuclease-Free Water, centrifuged at 14,000 rpm for 1 min, and controlled on agarose gel. Finally, the purified PCR product was sequenced by the Sanger methodology.

### 2.6. Digital Droplet PCR (ddPCR)

The absolute quantification of SARS-CoV-2-RNA was carried out by ddPCR using a two-step reaction; the cDNA synthesized using the SensiFAST cDNA Synthesis Kit (Bioline) was amplified using the 2× ddPCR Supermix (no dUTP) (Bio-Rad, Hercules, CA, USA). The QX200 droplet generator was used to generate the droplets by mixing the cDNA samples, 9 µM of both forward and reverse primers (reported in Table 4), and 2.5 µM of probe with 70 µL droplet-formation oil. The amplification step was performed on a T100 thermal cycler (Bio-Rad) with the following conditions: heat to 95 °C for 10 min, followed by 95 °C for 30 s, and 55 °C for 1 min, for a total of 45 cycles (at a heating rate of 2 °C/s), followed by 98 °C for 10 min. After the PCR, the positive/negative droplets were analysed in the QX200 droplet reader (Bio-rad), and the QuantaSoft analysis software (Bio-Rad) was used to calculate the number of targets analysed.

### 2.7. Confirmation of Subgenomic sgN and sgE Transcription in Vero E6 Cells

In order to confirm the levels of *sgE* and *sgN* in relation to those of the genomic regions amplified by the five kits, Vero E6 cells (8 × 10^5^) were infected with SARS-CoV-2 viral particles obtained from an Italian patient affected by COVID-19 (gene bank: MT682732.1), as previously reported [15]. After 72 h of infection, the total RNA was extracted from SARS-CoV-2–infected Vero E6 cells and reverse transcribed in cDNA [15]. The qPCR for *sgN*, *sgE* and *b-Actin* amplification was performed using SYBR Green chemistry. The quantity of the total viral RNA is expressed as Multiplicity of Infection (MOI): the latter was calculated by performing a standard curve with RNA related to an N1 fragment derived from standard positive controls (pcs) with a known viral titre (500000-50).

### 2.8. Running Conditions for Assay Comparisons

The iONEBIO and Gencurix plates were run on BioRad CFX instrumentation, following the manufacturers’ instructions. The remaining ones were all run on ABI7500 Dx fast and LC480-II Roche. On the latter, a specific updated software was used to decipher the results coming from each run. The threshold line values was set by default on the machine, and adjusted—when necessary—following the manufacturer’s indication. The specific threshold cycle (Ct) was used to distinguish the positive and negative samples, respectively.

### 2.9. Statistical Analysis

The statistical analyses were performed using the IBM SPSS^®^ Statistics (IBM Company, New York, NY, USA) software package (IBM SPSS Statistics for Mac, version 26). Correlation matrixes (Spearman rho coefficient) were used to show the linear relationship between the diagnostic tests. *p* ≤ 0.05 was regarded as being statistically significant. 

## 3. Results

**Figure 1:**
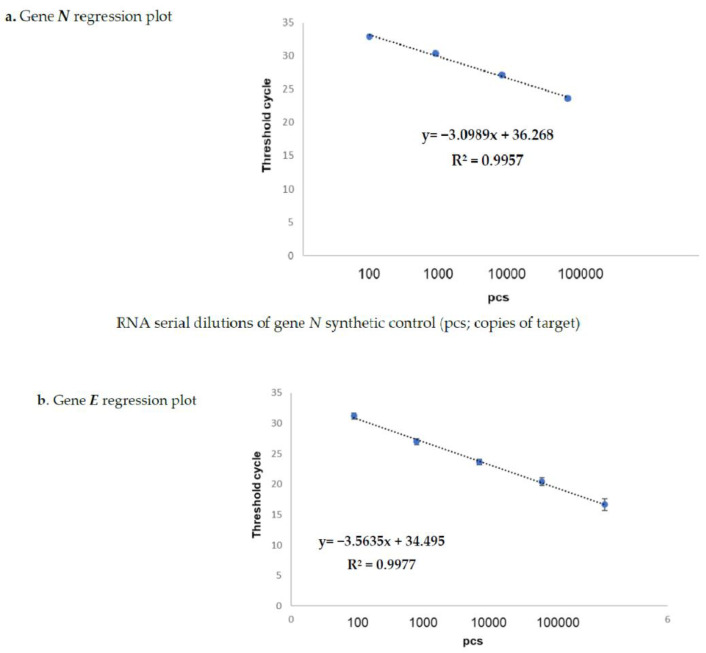
Spike-in of genes *N* (**a**) and *E* (**b**) evaluated by Kit-A. Figures (**a**,**b**) show the regression plots regarding the correlation curves obtained by the real-time TaqMan PCR Kit-A on serial dilutions of both synthetic positive control (pcs; copies of target) genea *N* and *E*. The starting volume of the input RNA for the reverse transcription and qPCR (one step process) was 5 µL for each target. The RNA amounts corresponding to each titration point amplified by one-step RT-qPCR were 0.1 pg, 1 pg,10 pg, 100 pg, and 1 ng for both the *N* and *E* genes, respectively. The reference sequences for the primers are reported in Table 3.

The results obtained on the sample dilutions of the spiked-in constructs are shown in Figure 1a,b, in which the amplification plots show an excellent evaluation of the regression linear coefficients (R2: 0.9957 and 0.9977) for *N* and for gene *E*, respectively, in triplicates assayed by means of the Allplex 2019-nCoV assay Seegene kit (KitA; Genova GE, Italy). The other kits reported in Table 2 also showed superimposable results. In fact, none of these failed in detecting the specific SARS-CoV-2 gene target at any dilution point (data not shown). The same results were also obtained on sampled spiked-in PBS buffer, therefore excluding the matrix effect during the extraction procedure (data not shown).

Therefore, following phase one, we analysed the RNA samples obtained from the collected from n.20 positive COVID-19 symptomatic patients. The positivity on these samples had been previously assigned by the use of kit-B and Kit-C. Here, in order to assess the reliability of RT-PCR, we tested the other three kits on the same RNA samples. As reported in Table 5, the kits showed different performances in terms of target detection (expressed as Ct values).

The viral E gene was included as a target in four of the kits, and the concordance of the detection rate was 100% (14/20 swabs), 75% (3/20), and 50% (3/20), respectively. For the RdRp gene, the concordance in the rate of detection was 100% in 15/20 swabs, and 66% in 5/20 samples. In 33% of cases, two out of the four kits missed the viral target. This behavior was also observed for the N gene, which was not detected in 5 out of 20 samples. The comparison of the gene ORF1ab performance was not evaluable due to absence of this target in the other commercial kits. Nevertheless, this target was not detectable in 6 out of 20 samples.

Then, in order to verify the superimposability of the results when they referred to the detection of the same viral genomic target (i.e., *N*, *E*, *RdRp*), correlation matrices were used. As reported in Table 6 and Table 7, by comparing three kits targeting gene E, the Genecurix method outperformed because of its significant correlation with the other two kits. The same behavior was observed for the ‘*RdRp*’ gene (Table 6).

Furthermore, we amplified the sgN and sgE targets, with the following results: only the four samples with a higher viral load (presence of mean Ct ≤ 22.5) and corresponding to patients with the most severe symptoms showed sgN positivity, while sgE showed positive results in all of the samples tested. These results, obtained on this explorative group of samples, were confirmed in two different runs, in duplicate, with a mean standard deviation of Ct values close to 0.1 Ct. Following the hypothesis that sgN is associated with higher virus load in the swabs, while sgE is not, we performed an in vitro experiment using Vero E6 cells [15] infected with the virus (Genebank MT682732.1) at different Multiplicities of Infection (MOI) (with standard positive controls of N1 gene, see Figure S2 and Table S1—ST1) following and verifying the expression of both sgN and sgE using SYBR Green real-time PCR. Our results unequivocally indicate that, while sgE is expressed at low levels (between 30.3 to 36.81 Ct) at the different MOI used (from 0.1 to 0.05), sgN is mainly expressed (between 25.36 to 33.72 Ct) on higher virus infected cells (MOI 0.15 to 0.02). The use of further dilutions of the virus (MOI < 0.02) corresponded to the undetectability of the sgN transcript (as reported in the Supplementary Table S1—ST1). Finally, in order to confirm these results in vivo in the swab, we retrospectively recovered n. 48 RNA samples (used as the discovery cohort) extracted from COVID-19 positive patients showing Ct values ranging from 13.5 to 22.5 (*n* = 26) to >22.5–40 (*n* = 22). The sgN target was detectable only in those samples with Ct values close to or below 22.5. In contrast, sgE was always detected in all of the Ct ranges. These results were obtained by both TaqMan and SYBR Green real-time PCR chemistries. Unfortunately, for these 48 retrospective samples, the contact tracing assessment of COVID-19 transmission was difficult to recover, particularly because we had received swabs from lots of different areas, and the centralized unit performing the swab sampling only reported these as symptomatic or asymptomatic. Consequently, it is difficult to correlate our confirmatory molecular results with the specific period of observation either for asymptomatic contacts or COVID-19 symptomatic individuals, even considering how hectic this swab collection activity has been. However, we can only report them as COVID-19–positive samples, and about 15% of these asymptomatic subjects showed low Ct values (<25.0).

Nonetheless, due to the recrudescence of COVID-19 in the last months, we were able to collect, in the first weeks of December, an additional 20 independent highly-positive samples (used as the validation group of samples), corresponding to: (a) four symptomatic individuals with severe forms of COVID-19 (mean Ct value ≤22.5); (b) six less severe patients with moderately-positive swabs (mean Ct ranging >22.5 and ≤29.5), and (c) 10 asymptomatic subjects corresponding to the close contacts of infected patients who were screened for the first time, and submitted to a swab drawing one week after the onset of symptoms in the positive individuals (n. 4, with a mean Ct value ≤22.5; n. 4 with mean Ct > 22.5–29.5, and n. 2 with mean Ct > 29.5), where we confirmed the above reported data. Furthermore, in order to exclude false positive results given by any possible off-target signal, we used high resolution melting analysis (HRMA) and Sanger sequencing.

**Figure 2:**
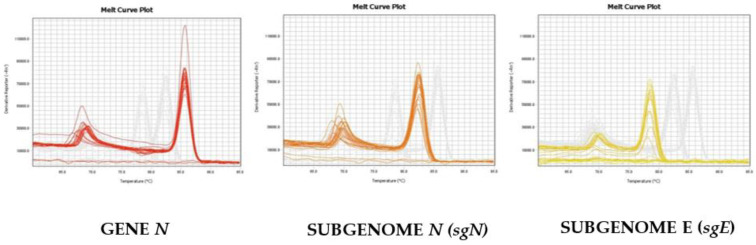
Melting curve profiles obtained by HRMA on the different genomic and subgenomic regions of SARS-CoV-2. The amplification plots of the three targets are clearly shown, as the melting temperatures (Tm) are different: Gene *N* (Tm = 86.5), sgN (Tm = 82.5), and *sgE* (78.0), respectively.

As reported in Figure 2, we were able to distinguish and confirm the different targets amplified by HRMA. In order to better validate our results, we sequenced the sgN region, obtaining the following amplicon of 141, as reported in Figure S3. Data regarding subgenomic sequencing by Nanopore technology of the subgenomic RNAs of SARS-CoV-2 have been published by our group [15]. The virus that was isolated, fully sequenced, and used in our studies has a GeneBank number of MT682732.1 (Severe acute respiratory syndrome coronavirus 2 isolate SARS-CoV-2/human/ITA/Naples/2020, complete genome). The GISAID annotations for all of the genomic and subgenomic regions are reported in the Ref [15].

**Figure 3:**
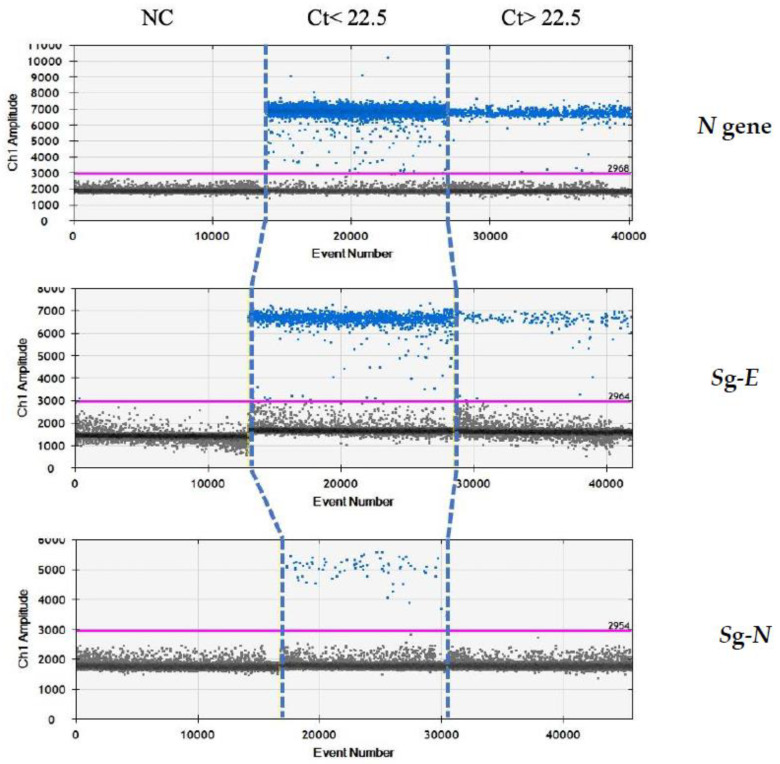
Analysis by ddPCR analysis on samples with low Ct (≤22.5) for *N*, *sgE* and *sgN transcripts*. ddPCR analysis output regarding samples with either Ct ≤ 22.5 or Ct > 22.5 in the realtime PCR analysis. Gene *N* is present in all of the samples with Ct below or above the 22.5 value. The *sgN* RNA is only detectable for Ct ≤ 22.5, while the *sgE* transcript is within all of the of the Ct ranges.

Finally, we re-analysed these samples by droplet-digital-real-time PCR (ddPCR) in order to evaluate whether the absence of *sgN* in the samples above Ct 22.5 was due to the limit of detection of the real-time methods, rather than being due to the characteristics of samples containing a lower viral load. Our ddPCR results (reported in Figure 3) clearly show that *sgE* is detected in samples with Ct > or ≤22.5 (from 13.5 to 40), while the *sgN* is detected only when Ct was ≤22.5. It is of interest that the *sgN* expression does not seem to be influenced by genomic *N* gene transcription, with the *N* gene being detectable in all of the Ct ranges (from 13.5 to 40.0). 

## 4. Discussion

In the present report, we provide extensive data regarding an evaluation detection of subgenomic transcripts in relationship to the positivity rate (evaluated as Ct values) of three CE-IVD commercial kits used during the first outbreak of COVID-19 pandemic, plus two new kits recently released onto the market. The use of these different reagents—alternatively or as a complementary, especially in the case of uncertain results—was necessary to verify not only the relative performances, but also to correlate the amplification rate of *N*, *E*, and *RdRp* targets with the subgenomic expression of the *sgN* and *sgE* transcripts. In fact, it is reported that the amplification of the complete SARS-CoV-2 genome is possible only when the threshold cycle is below 33.0. Due to both the preanalytical and analytical variables [5,16] affecting the virus detection in oro-nasopharyngeal swabs, we used different methods in order to better evaluate the expression of the subgenomic transcripts in relationship to the genomic transcripts. The high-sensitivity PCR-based tests are nearly 100% accurate in identifying infected people, if they are administered appropriately [1,17]. In order to avoid biases related to the amplification process of RNA from unknown swabs, we evaluated the analytical performance of the five kits reported herein using both negative sample swabs and phosphate buffer medium, into which genomic constructs of gene *N* and *E* were spiked-in at different dilutions. In these theoretically-ideal conditions (the certainty that at least a SARS-CoV-2 RNA target was present), at the different dilutions, we obtained the result that all of the kits were able to detect the target within the limit of detection range declared. This means that both our automated extraction method and the medium used for the dilutions (TMB or PBS) did not influence either the yield or the recovery of the *N* and *E* artificial targets spiked in at the different concentrations. Thus, we excluded the presence of any possible interference by the matrix effect. Nevertheless, the same findings were not so exciting when they referred to the twenty positive samples contemporarily analyzed with the five alternative methods: in fact, in no case have we obtained a complete superimposition of the results, with a correlation from 50% to 100% among the different commercial kits. We could hypothesize that this lack of correlation depends either on the viral load or on the type of components within each reagent. Moreover, we did not observe any signal related to probe degradation or increases in the background signals within each microplate. Consequently, we can only report the discrepancies among the different methods evaluated as the results of different sensitivities on the true clinical samples as compared to those obtained on the spiked-in materials. The best correlation matrix was found for GenePro COVID-19 (Kit-E), which was shown to be the kit with the best performances in terms of both target detection and concordance with the tools targeting the same regions of SARS-CoV-2. This commercial kit was also reported by Kubina R. et al. [18] as being superimposable to other commercial CE-IVD assays. We underline that one important feature of the SARS-CoV-2 diagnostic path is to anticipate—as soon as possible—its detection in order to rapidly screen the population and avoid or limit its spread. Unfortunately, in the presence of the tools and reagent not overlapping in terms of target detection, it would be very difficult to distinguish people who are under an active viral infection. In this regard, Guglielmi G. et al. [10] underlined the fact that it is crucial to identify the infected individuals who are not able to spread the virus as only ‘passive’ carriers. In order to overcome the limits given by the kits targeting the SARS-CoV-2 genomic transcripts, we wanted to test whether other molecular markers of SARS-CoV-2 could be detectable as also being able to describe the kinetics of the virus replication inside each individual. Therefore, within the complex replicative machine of SARS-CoV-2, we designed a specific assay to amplify the *sgN* and *sgE*, which correspond to the subgenomic regions, within positive swabs. Surprisingly, we found that *sgN* mRNA is transcribed only in the samples with the highest viral loads, as extrapolated by the Ct values, regardless of the kit that is routinely used. In fact, the *sgN* detection was achievable whenever the mean Ct value of the run is below or equal to 22.5. These findings were further validated in vitro with different virus load on Vero E6 cells, in which the *sgN* was highly expressed in samples where the genomic targets *N* and *E* showed the lower Ct. Then, a further confirmation of our results was also obtained by ddPCR, which did not detect any *sgN* copy in any of the samples below the 22.5 value, suggesting that *sgN* could be further investigated as a reliable marker of the highest viral load on a larger sample number. Our results, confirmed in all of the eighty-eight swab samples analyzed, therefore do not depend on the limit of detection of our assays being ddPCR more sensitive that real-time PCR. These results appear not to be in contrast with previous experimental findings [7,11,12] showing that the *N* protein is required for efficient coronavirus subgenomic mRNA transcription. In order to confirm our hypothesis of a potential use of subgenomic transcripts in molecular diagnostics, we confirmed in vitro*,* using Vero E6 cells with different MOI virus infection rates, that *sgN* was detectable every time the viral load was highly evaluated. These results were not obtained when *sgE* was analyzed. Regarding the behavior of *sgE*, we verified that this target is expressed in all of the samples, regardless of the individual Ct value of the specific genomic targets amplified by the different kits. Therefore, we could speculate that *sgN* is a surrogate marker of both the highest viral load and potential infectivity, although this result needs to be confirmed on a standardized cohort of patients, with a precise indication of the infection time, severity of symptoms, and clinical and molecular evaluation during the follow-up. We highlight here that the mechanism for the generation of these subgenomic mRNAs is not fully understood, although they are strongly regulated to guarantee the best ratio of virus proteins and their survival in the cells. Furthermore, the expression of the N protein seems to be required for an efficient coronavirus subgenomic mRNA transcription, as confirmed by the detection of high numbers of copies of this gene by ddPCR: the N protein stabilizes, in fact, the virus genome copies once it is replicated into the cell.

We further underline that subgenomic RNAs are considered to be particularly abundant during the early infection (up to 70 times more abundant than virus genomic RNA at the peak of RNA transcription in the cell culture), as it occurs as early as 6-8 h after infection [7,12]. The detection of these subgenomic RNAs in our clinical nasopharyngeal swabs confirms that these transcripts are moderately stable, being the *sgN* RNA mainly associated to the highest viral load. Moreover, in contrast to the data reported by Alexandersen et al. [17], who reported that the relative abundance of subgenomic RNAs may be more related to the sample’s quality and storage before the laboratory assay than to the actual stage of infection, we can emphasize that the persistence of *sgN* RNAs might be considered as a candidate biomarker of the active viral load, regardless of the pre-analytical conditions and analytical diagnostic kit. It is noteworthy that *sgN* was reported as the most expressed transcript in other sample types, like stool [19]; the authors of this research concluded that the detection of *sgN* and s*gE* improves the diagnosis of COVID-19, particularly in patients who are suspected of being infected but with negative results in the upper respiratory tract. Although the setting of this study [19] is different, we can agree with the authors’ conclusion, which emphasized the role of these subgenomic transcripts. Our preliminary findings, although they were confirmed on a group of eighty-eight samples, should be deepened on larger cohorts, particularly to confirm the diagnostic potential of the *sgN* target in terms of the prediction of higher and active viral loads. Nevertheless, it is not so common to find clinical swabs with these high viral loads, as confirmed—in our experience—on more than 70,000 swabs processed in the last six months, for which the recovery of samples with very high viral loads (intended as with a Ct ≤22.5) is not very frequent. Overall, we recovered about sixty samples with Ct ≤22.5 in the March–October pandemic infection period. We point out that the main limitation of our study is that we were not able to precisely trace the timing of the infection of the individuals who submitted to oro-nasopharyngeal swabs, particularly for the samples collected outside of our hospital. In this regard, we agree with Cheng et al. [20], who reported that the dynamics of COVID-19 transmissibility are far from being fully understood. However, it is important to investigate possible new markers for a better understanding of the transmission dynamics for the development and evaluation of effective control policies. Cheng et al. [20] reported that the short serial interval of COVID-19, and the results from viral shedding studies indicate that most transmission occurred near—or even before—the time of the onset of symptoms. Nevertheless, it is not known when and for how long the individual with COVID-19 should be isolated, or whether close contacts should be quarantined. Therefore, we emphasize that every study can be affected by these pre-analytical biases.

However, Alexandersen et al. [17] stated that SARS-CoV-2 subgenomic RNAs were detectable in about thirteen diagnostic samples up to 17 days after the initial detection of infection. This evidence seems to be related to their nuclease resistance and protection by cellular membranes. Nevertheless, they performed an untargeted analysis in all of the subgenomic regions, which was able to amplify some subgenomic RNAs by NGS, using various amplification steps to amplify the short targets. This approach, on a limited number of samples, cannot be superimposable to our setting, in which we employed different methodologies in an unbiased bioinformatic method of nucleotide leader sequence selection. Therefore, based on our findings, we do not agree with their conclusions assessing that the detection of subgenomic RNAs in clinical samples may not be a suitable indicator of active coronavirus replication/infection.

Thus, we show here results underlining that *sgN* positivity can unequivocally reflect a stage at the highest viral load, discriminating between more active (early) virus infection and middle–long-term carrier status. This hypothesis is not in contrast with findings by Wölfel et al. [12], who reported that “viral subgenomic mRNA is transcribed only in infected cells indicating the presence of actively infected cells in samples”. Moreover, the capability of the virus to grow, particularly when it is isolated from samples with lower Ct values and *sgN* detectability, has been demonstrated by our group in a parallel research work that is under publication elsewhere. These findings seem to be in contrast with those published by Kim et al. [11], who found that the *N* RNA is the most represented during the viral replication; nevertheless, these data were obtained from SARS-CoV-2 viral RNA extracted from Vero cells infected with BetaCoV/Korea/KCDC03/2020, the latter being an experimental setting that was different from ours. In support of our considerations, we can provide evidence by Winnett et al. [21] stating that low-sensitivity tests are comparable to high-sensitivity tests in detecting early infections when the viral load rises quickly (within hours) after infection and reaches high levels (>10^5^–10^6^ RNA copies/mL). However, although there are no human data testing these assumptions, they provided evidence that, in at least some human cases of SARS-CoV-2, the viral load rises slowly (over days, not hours) and not to such high levels as to be detectable reliably by any low-sensitivity test. We showed herein that our commercially-available kits fail in detecting some SARS-CoV-2 genomic targets in swab-deriving RNA; therefore, the use of subgenomic transcripts might improve the rate of virus detection.

This evidence is of impact because it could be mainly useful during follow-ups in which the positivity of the molecular test cannot discriminate between higher or lower viral loads, while the clearance of SARS-CoV-2 from the respiratory tract occurs, sometimes asymptomatically. 

We encourage other laboratories providing SARS-CoV-2 assays to further investigate in this setting, because conflicting findings around reinfection, as well as discrepancies among diagnostic PCRs detecting targets in different regions of the SARS-CoV-2 genome, are reported in the literature, mostly when they are related to subgenomic transcripts.

## 5. Conclusions

This is the first paper showing data on *sgN* transcript detection in nasopharyngeal swabs of SARS-CoV-2 infected individuals with higher viral loads (low Ct values). Our findings, sometimes in contrast with those reported in vitro [11], when translated to the in vivo context, may reflect the not-completely-known aspects regarding the biology of the virus within the nasopharyngeal trait [22]. Many individual habits could have influenced the viral load, particularly mouth rinsing [23]; as such, in vitro and in vivo results are not always superimposable, as most of the mouth rinses tested were able to inactivate SARS-CoV-2 viruses. Up to now, no study has definitively measured the duration of infectivity [24]; however, patients may not be infectious for the entire duration of the detection of the virus, as the presence of viral ribonucleic acid may not represent transmissible live virus [24]. Some differences in mRNA viral recovery were found depending on the starting biological sample (sputum, saliva, upper respiratory tract samples, or stools) [24]. Therefore, we will evaluate, in the future, other possible strategies for the early detection and monitoring of SARS-CoV-2 in other patients’ samples. Nevertheless, we cannot exclude that individual behaviors have influenced the rate of recovery of subgenomic RNAs. Therefore, we could only assume that our findings might be due to: (i) the shorter half-life time of *sgN*, as demonstrated by its non-detectability by ddPCR in the lower viral loads; (ii) the preferable isolation of *sgE* over *sgN*; (iii) the association of sgN with the presence of replicative intermediates of the virus that could justify its higher stability and presence during the more active infection/infectivity time. Further studies evaluating these aspects are necessary in order to better explain the above hypotheses. 

## Figures and Tables

**Table 1:** List of reagents indicated by Italian Istituto Superiore di Sanità as the recommended diagnostic tools during the first pandemic period (March 2020).

Reagent/Kit Name	Company
Bosphore Novel Coronavirus (2019-Ncov) Detection Kit (Kit-B)	Anatolia Tani Ve Bijotecknoloji Urunleri Arastirma Gelistirme Sanaji Ve Ticaret Anonim Sirketi
STANDARD M nCoV Real-Time Detection Kit	SD Biosensor Inc
Allplex 2019-nCoV assay (Kit-A)	Seegen Inc.
QUANTY COVID-19	CLONIT SRL
GENEFINDER COVID-19 PLUS REAL-LAMP KIT	OSANG HEALTHCARE
NOVEL CORONAVIRUS (2019-NCOV) REAL TIME MULTIPLEX RT-PCR KIT	SHANGHAI ZI BIO-TECH CO., LTD
ON-SITE RAPID PCR DIAGNOSTIC SYSTEM	SOSEPHARM
LABGUN COVID-19 ASSAY	LABGENOMICS CO. LTD.
REALQUALITY RQ-2019-NCOV (Kit-C)	AB ANALITICA SRL
CORONA VIRUS DISEASE 2019 (COVID-19) NUCLEIC ACID DETECTION KIT	OACP S.R.L.
SIMPLEXATM COVID-19 Direct assay	DiaSorin Molecular LLC

**Table 2:** Characteristics of the five kits used for the detection of SARS-CoV-2 genomic targets.

	Parameters	Kit-A	Kit-B	Kit-C	Kit-D °	Kit-E
**GENES**	**Gene E**	x	x	x		x
	**RdRP**	x		x		x
	**Gene *n***	x			x	
	**orf1ab**		x			
**INSTRUMENTS**	**ABI 7500**		x	x		x
	**LC 480**		x	x		
	**Montania**		x			
	**BioRad CFX96Dx**	x	x	x	x	x
	**Rotorgene 6000**		x			
	**Q-Qiagen**		x			
	**Atila PG 9600**					
	**ABI QuantStudio 5DX**					
	**AriaDx**			x		
	**Mic**			x		
**KIT FEATURES**	**LoD**	100 copies/rxn	25 copies/rxn	3 copies/rxn	1 copie/rxn	50 copies/rxn
	**Precision**	CV ≤ 5%	not declared	CV ≤ 5%	not declared	CV ≤ 5%
	**Treshold Ct**	≤40 positive ≤40 IC	≤30 PC ≤32 IC	≤34 IC	≤25 positive	≤40 positive
	**Number of total cycles**	45	35	45	35	45
	**Time**	~1 h 30 min	~1 h	~1 h 30 min	20 min	~1 h 30 min
	**RNA Volume**	8 μL	10 μL	10 μL	5 μL	5 μL OM1 5 μL OM2
	**Final Volume of reaction**	25 μL	25 μL	30 μL	25 μL	25
	**Volume of Internal** **Control**	10 μL in the starting sample	5 μL when starting with extraction; 0.2 μL when spiked in RNA	1 μL in 6 µL elution volume	Not included	Not included

° Ionebio (Kit-D; http://www.ionebio.com (accessed on 4 February 2021)) is an isothermal-based method which determines as positive all samples with a Cycle thershold (Ct) ≤ 25. This kit works as a loop-mediated isothermal amplification (LAMP) method; CV: coefficient of variation; IC: internal control; OM: mastermix; PC: positive control. Kit E: https://www.theglobalfund.org/media/9629/covid19_diagnosticproducts_list_en.pdf (accessed on 4 February 2021).

**Table 3:** Primers for sgRNA detection by real-time PCR.

Target Viral Gene	Primer	Primer Sequence	Reference Regions (5′-3′ Nucleotides)
***Sg-N*** [7,14] **(TaqMan)**	Forward	CAACCAACTTTCGATCTCTTGTA	18–40
	Reverse	TCTGCTCCCTTCTGCGTAGA	28771–28790
	Probe	5′FAM-ACTTCCTCAAGGAACAACATTGCCA-BBQ-3′	28738–28763
***SYBR-Green***	Forward	*CAAACCAACCAACTTTCGATCTCTTGTA*	12–40
	Reverse	*TCTGGTTACTGCCAGTTGAATC*	29669–29693
***Sg-E*** [12] **(TaqMan)**	Forward [1]	CGATCTCTTGTAGATCTGTTCTC	29–51
***SYBR-Green***	Reverse [1]	ATATTGCAGCAGTACGCACACA	26346–26367
	Probe [1]	5′FAM-ACACTAGCCATCCTTACTGCGCTTCG-BBQ-3′	26318–26343
	Forward	*CAAACCAACCAACTTTCGATCTCTTGTA*	12–40
	Reverse	*AGAAGTACGCTATTAACTATT*	26266–26287
**N_*gene*** [7] **(TaqMan)**	Forward	CACATTGGCACCCGCAATC	28692–28800
	Reverse	GAGGAACGAGAAGAGGCTTG	26346–26367
	Probe	5′FAM-ACTTCCTCAAGGAACAACATTGCCA-BBQ-3′	28738–28763
***SYBR-Green***	Forward	*GACCCCAAAATCAGCGAAAT*	28273–28293
	Reverse	*TCTGGTTACTGCCAGTTGAATCTG*	28321–28345

**Table 4:** Primers used for the Spike generation constructs.

RNA Oligonucleotide	Sequence Targeted Regions
**SARS-CoV-2_N_F1** (28260–28376)	AUGUCUGAUAAUGGACCCCAAAAUCAGCGAAAUGCACCCCGCAUUACGUUUGGUGGACCCUCAGAUUCAACUGGCAGUAACCAGAAUGGAGAACGCAGUGGGGCGCGAUCAAAACAA
**SARS-CoV-2_E** (26255–26376)	ACAGGUACGUUAAUAGUUAAUAGCGUACUUCUUUUUCUUGCUUUCGUGGUAUUCUUGCUAGUUACACUAGCCAUCCUUACUGCGCUUCGAUUGUGUGCGUACUGCUGCAAUAU

Notes: in parenthesis are reported the reference regions targeted in terms of 5′–3′ nucleotides.

**Table 5:** Comparisons of the performances of each kit, referring to the different targets detected and reported as the threshold cycle (Ct) in Covid19-positive swabs.

Sample ID	SEEGENE (Kit-A)			ANATOLIA (Kit-B)		AB-ANALITICA * (Kit-C)		IONEBIO (Kit-D)	GENCURIX (Kit-E)		Concordance (%) on the Target		
	Gene *N*	Gene *E*	Gene *RdRp*	Gene *ORF*1*ab*	Gene *E*	Gene *RdRp*	Gene *E*	Gene *N*	Gene *RdRp*	Gene *E*	Gene *E*	Gene *RdRp*	Gene *N*
	(Ct)	(Ct)	(Ct)	(Ct)	(Ct)	(Ct)	(Ct)	(Ct)	(Ct)	(Ct)	%	%	%
**M3**	33.18	30.26	31.68	24.02	24.05	23.2	22.8	3.96	23.87	24.35	100	100	100
**R**	22.46	18.81	19.78	22.1	22.2	21.6	21.5	4.18	22.19	23.18	100	100	100
**S**	23.42	20.92	22.44	26.03	26.06	27.1	26.5	4.04	25.15	25.7	100	100	100
**S3**	19.75	16.03	17.43	19.2	19.03	19.2	18.9	3.91	19.82	20.24	100	100	100
**T1**	23.58	21.67	22.67	25.3	25.1	25.1	24.8	9.29	25.44	26.0	100	100	100
**D**	33.18	30.26	31.68	ND	ND	33.6	33.1	10.78	39.84	34.63	75	100	100
**I3**	33.19	29.6	32.25	34.05	34.03	34.1	33.6	**26.04**	35.4	35.5	100	100	50
**V2**	34.75	31.19	37.02	ND	ND	34.6	34.1	25.0	ND	36.66	75	66	100
**E2**	21.67	17.54	18.66	22.08	22.05	22.03	22.2	3.73	20.41	21.23	100	100	100
**C3**	30.49	28.09	29.65	32.07	32.	32.01	32.3	**25.99**	31.85	32.28	100	100	50
**G3**	33.63	30.42	31.92	ND	ND	34.04	34.1	16.64	ND	ND	50	66	100
**L3**	24.98	21.51	23.35	26.04	26	26.02	26.2	14.93	24.94	25.2	100	100	100
**6**	27.23	28.59	28.12	24.05	23.3	26.3	26.1	3.67	26.23	27.21	100	100	100
**7**	23.17	24.56	23.92	22.51	22.07	22.9	22.8	**32.66**	24.52	25.4	100	100	50
**12**	30.13	32.04	30.23	25.41	25.11	30.7	30.3	3.23	29.17	30.34	100	100	100
**24**	35.18	38.54	35.27	ND	ND	35.5	35.5	2.88	ND	37.23	75	66	100
**145**	37.34	38.18	36.51	ND	ND	34.7	34.1	3.22	ND	ND	50	66	100
**146**	29.04	31.11	29.16	24.48	24.5	28.2	27.02	3.03	28.72	29.33	100	100	100
**149**	35	36.81	34.25	ND	ND	34.7	33.9	ND	ND	ND	50	66	50

Kits compared and reported in Table 5: Ionebio; Allplex 2019-nCov Assay (SEEGENE); BOSPHORE Novel Coronavirus 2019-Ncov (ANATOLIA); RealQualityRQ-2019-NCOV (AB-ANALITICA, * = the reference method); GenePro COVID-19 GENCURIX; ND = not detected. The data marked ‘ND’ were treated as missing values of detection by the kit, and excluded/ignored from the correlation reported in Table 6 and Table 7. In detail, SPSS uses pairwise deletion of missing values: each correlation uses all of the cases having valid values for all of the of the variables.

**Table 6:** Correlation matrix of three commercial kits targeting gene *E* and *RdRP*.

Gene E		Kit-A	Kit-B	Kit-C	Kit-E
**Kit-A**	Spearman’s rho	-	-	-	-
	*p*-value	-	-	-	-
**Kit-B**	Spearman’s rho	0.445	-	-	-
	*p*-value	0.13	-	-	-
**Kit-C**	Spearman’s rho	0.828	0.894	-	-
	*p*-value	≤0.001	≤0.001	-	-
**Kit-E**	Spearman’s rho	0.808	0.769	0.962	-
	*p*-value	≤0.001	0.003	≤0.001	-

**Table 7:** Correlation matrix of three commercial kits targeting gene RdRp.

Gene RdRp		Kit-A	Kit-C	Kit-E
**Kit-A**	Spearman’s rho	-	-	-
	*p*-value	-	-	-
**Kit-C**	Spearman’s rho	0.899	-	-
	*p*-value	≤0.001	-	-
**Kit-E**	Spearman’s rho	0.772	0.965	-
	*p*-value	≤0.001	≤0.001	-

## Data Availability

Please refer to www.ceinge.unina.it (accessed on 4 February 2021).

## Supplementary Materials

The following are available online at https://www.mdpi.com/2075-4418/11/2/288/s1. Figure S1: Primer setting for *N gene* and *sgN* regions. Figure S2: Gene *N* regression plot and gene expression values for *sgN* and *sgE* (see Table S1). Table S1 (ST1): SYBR Green qPCR showing Ct and ΔCt values related to sgN and sgE transcripts on RNA extracted from Vero E6. Figure S3: Sanger sequencing of the *N* subgenomic region of SARS-CoV-2.
