# Supplementary material for: SARS-CoV-2 Subgenomic N (sgN) Transcripts in Oro-Nasopharyngeal Swabs Correlate with the Highest Viral Load, as Evaluated by Five Different Molecular Methods
Source: Diagnostics (Basel). 2021 Feb 12;11(2):288. doi: 10.3390/diagnostics11020288 (PMC7923082; doi:10.3390/diagnostics11020288)
Supplement: Supplementary file 1 [file diagnostics-11-00288-s001.pdf]

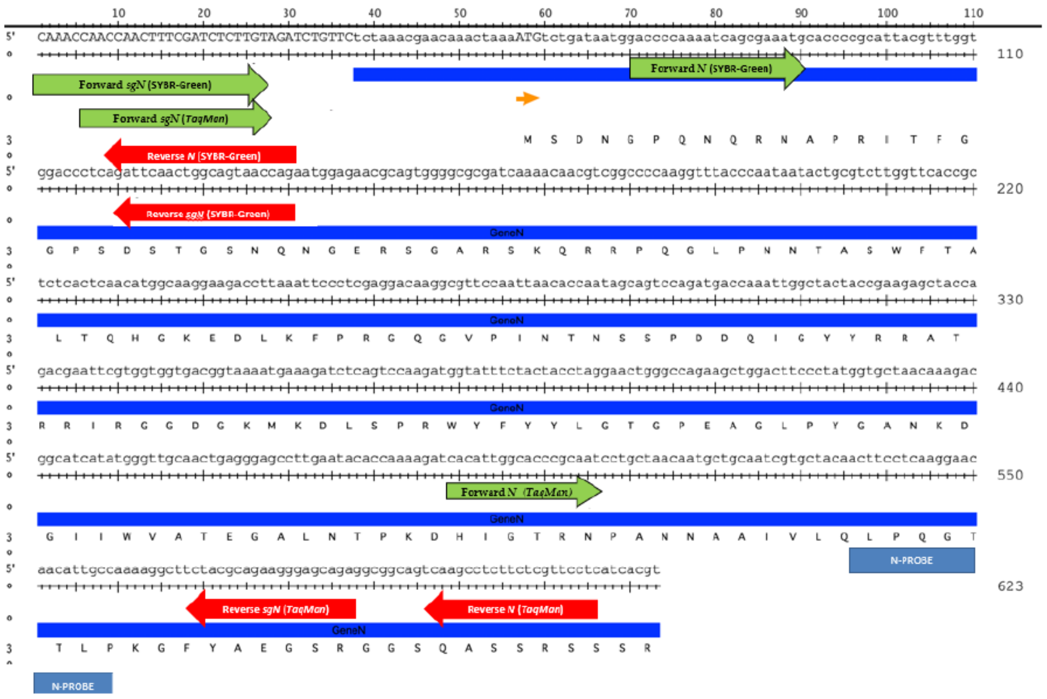

Figure S1. Primer setting for *N gene* and *sgN* regions. In green are reported the Forward primers for genomic and subgenomic transcripts of *N* gene. In red, the Reverse ones. In light blue the common probe is indicated.

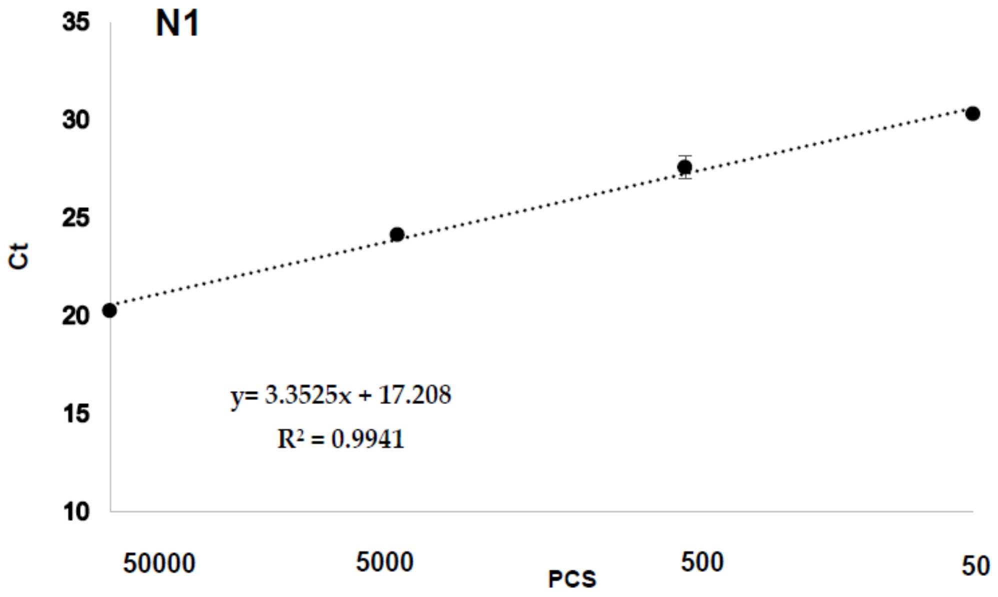

Figure S2. Gene *N* regression plot and gene expression values for *sgN* and *sgE*. Standard curve with RNA related to N1 fragment derived from synthetic positive controls (pcs) with a known viral titre (500.000–50). The "Y" axes bar shows threshold cycle (Ct) related to N1 amplification obtained with SYBR Green approach; on the "X" axes RNA serial dilution from Synthetic Positive Control (pcs**)**

Table S1 (**ST1**): SYBR Green qPCR showing Ct and ΔCt values related to *sgN* and *sgE* transcripts on RNA extracted from Vero E6.

SARS-CoV-2 Infected Vero E6 cells				
**MOI**	***sgN***		***sgE***	
	**Ct**	**ΔCt**	**Ct**	**ΔCt**
**0.15**	**25.36**	**8.33**	**30.33**	**13.31**
**0.10**	**31.48**	**12.69**	**35.26**	**16.48**
**0.09**	**31.25**	**11.76**	**34.18**	**14.68**
**0.02**	**33.72**	**15.84**	**36.05**	**18.17**
**0.01**	**N.D.**	**N.D.**	**39.62**	**22.08**
**0.03**	**N.D.**	**N.D.**	**36.52**	**18.16**
**0.05**	**N.D.**	**N.D.**	**36.81**	**17.56**

Legend Table ST1: qPCR with SYBR Green approach showing Ct and ΔCt values related to sgN and sgE transcripts on reverse transcribed RNA extracted from Vero E6 cells infected with SARS-CoV-2 at different Multiplicity of Infection (MOI). ΔCt values were calculated by using b-Actin as housekeeping gene.

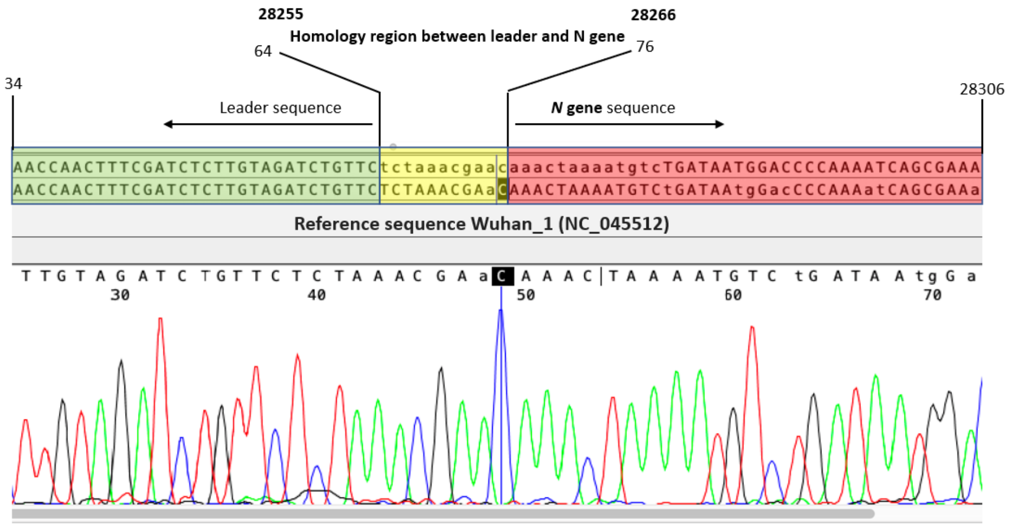

Figure S3. Sanger sequencing of *N* subgenomic region of SARS-CoV-2. The primer pair that recognizes sequences on the virus genome 28331 nucleotides distant, when used to amplify cDNA from swabs, produces a PCR amplicon of 141 bp. The region of homology between the leader and *N* gene is highlighted in yellow [15]. The arrows highlight the fusion between the leader and *N* gene sequences, respectively. The Sanger sequencing of this PCR product results in the *sgN* sequence.
